# Seasonal Phenological Patterns and Flavivirus Vectorial Capacity of Medically Important Mosquito Species in a Wetland and an Urban Area of Attica, Greece

**DOI:** 10.3390/tropicalmed6040176

**Published:** 2021-09-28

**Authors:** Stavroula Beleri, Georgios Balatsos, Vasilios Karras, Nikolaos Tegos, Fani Sereti, Georgios Rachiotis, Christos Hadjichristodoulou, Nikolaos Papadopoulos, Dimitrios Papachristos, Antonios Michaelakis, Eleni Patsoula

**Affiliations:** 1 Department of Public Health Policy, School of Public Health, University of West Attica, 115 21 Athens, Greece; ntegos@uniwa.gr (N.T.); epatsoula@uniwa.gr (E.P.); 2 Scientific Directorate of Entomology and Agricultural Zoology, Benaki Phytopathological Institute, 145 61 Attica, Greece; g.balatsos@bpi.gr (G.B.); v.karras@bpi.gr (V.K.); d.papachristos@bpi.gr (D.P.); a.michaelakis@bpi.gr (A.M.); 3 School of Public Health, University of West Attica, 115 21 Athens, Greece; sereti.fani@gmail.com; 4 Department of Hygiene and Epidemiology, University of Thessaly Medical School, 412 22 Larissa, Greece; grach@uth.gr (G.R.); xhatzi@uth.gr (C.H.); 5 Department of Agriculture, Crop Production and Rural Environment, University of Thessaly, 38 446 Volos, Greece; nikopap@uth.gr

**Keywords:** Culex, BG-sentinel, pathogens, fixed sampling site, RT-PCR

## Abstract

Seasonal patterns of mosquito population density and their vectorial capacity constitute major elements to understand the epidemiology of mosquito-borne diseases. Using adult mosquito traps, we compared the population dynamics of major mosquito species (*Culex pipiens*, *Aedes albopictus*, *Anopheles* spp.) in an urban and a wetland rural area of Attica Greece. Pools of the captured *Cx. pipiens* were analyzed to determine infection rates of the West Nile virus (WNV) and the Usutu virus (USUV). The data provided were collected under the frame of the surveillance program carried out in two regional units (RUs) of the Attica region (East Attica and South Sector of Attica), during the period 2017–2018. The entomological surveillance of adult mosquitoes was performed on a weekly basis using a network of BG-sentinel traps (BGs), baited with CO_2_ and BG-Lure, in selected, fixed sampling sites. A total of 46,726 adult mosquitoes were collected, with larger variety and number of species in East Attica (n = 37,810), followed by the South Sector of Attica (n = 8916). The collected mosquitoes were morphologically identified to species level and evaluated for their public health importance. Collected *Cx. pipiens* adults were pooled and tested for West Nile virus (WNV) and Usutu virus (USUV) presence by implementation of a targeted molecular methodology (real-time PCR). A total of 366 mosquito pools were analyzed for WNV and USUV, respectively, and 38 (10.4%) positive samples were recorded for WNV, while no positive pool was detected for USUV. The majority of positive samples for WNV were detected in the East Attica region, followed by the South Sector of Attica, respectively. The findings of the current study highlight the WNV circulation in the region of Attica and the concomitant risk for the country, rendering mosquito surveillance actions and integrated mosquito management programs as imperative public health interventions.

## 1. Introduction

Research on the distribution, abundance, and species composition of mosquitoes at a regional level is vital to estimate the risk of incidence of vector-borne diseases that are currently increasing in Europe, because of range expansion of native species and invasion events by alien species [1,2,3,4]. Factors, such as globalization of travel and trade, increasing land use and urbanization, high concentration of human populations, socioeconomics, and climate change, enhance viral circulation. As a result, invasive species have expanded considerably their geographical and vectorial range, therefore increasing the risk of human exposure [5,6,7].

Entomological studies conducted in many regions of Greece [8,9,10,11,12,13,14] and in other European countries [15,16,17] demonstrate the importance of mosquito surveillance for transmitted viruses that can be a powerful tool as a part of an effective early-warning system [10,12,14,18,19]. Testing Culex mosquitoes for WNV, especially in high-risk areas is helpful for gaining insight into the virus circulation; it is a significant confirmation in cases where WNV was detected in mosquitoes before the symptoms’ onset in the human cases [14,20,21].

The emergence and resurgence of certain mosquito-borne diseases has led to the implementation of integrated programs, including entomological, veterinary, and human surveillance in several European countries. The goal is to prompt recognition and monitoring of arboviral activity; hence, the activation of proper control measures to prevent transmission [3,5,22,23,24].

Among several arboviruses being endemic in Europe and the Mediterranean Basin, two neurotropic mosquito-borne flaviviruses, the West Nile virus (WNV) and the Usutu virus (USUV), belonging to the Japanese encephalitis antigenic complex [25,26], cause sporadic cases of infection and outbreaks during the transmission seasons [5,13,14,27,28].

West Nile virus (WNV, Flaviviridae) is amongst the most widespread flavivirus in the world [29,30]. Since its discovery in 1937, it has spread beyond its original known geographic range and has caused human disease on every continent except Antarctica. It is continuously circulating in Europe with a recent increasing trend of incidence in several countries [3,31,32]. Greece is one of the most WNV-affected European countries with outbreaks of the virus being recorded since 2010 [13,14,18,30,33,34,35,36]; since then, the cases of WNV remain high on an annual basis [37,38,39].

The less renowned Usutu virus (USUV, Flaviviridae) is an African mosquito-borne flavivirus [40,41,42] that constitutes a worrisome threat to human and animal health worldwide. The virus was first detected in South Africa in 1959 [43,44], with the first cases in Europe dated in 1996 [45,46,47]. The first USUV outbreak was recorded in Austria in 2001 [27,48,49], and since then, the virus has spread throughout Europe [44,50,51], causing a considerable mortality among bird populations [50,52,53,54] and creating increasing concern for the potential zoonotic transmission to humans [40,55,56,57,58,59,60,61]. Usutu virus antibodies were first detected in Greece in 2010 in a dove [50,62], but no human cases have ever been recorded [10]. Nevertheless, targeted surveillance programs for vectors were not implemented; so far, both viruses share a similar enzootic transmission cycle, with birds as amplifying hosts and ornithophilic mosquitoes as vectors [5,50,51], and there are cases where the two viruses co-circulate in the same environment [17,51]. It has been reported that co-circulation of USUV and other related Flavivirus-like WNV may have an impact in terms of the respective epidemiological mode [28].

In this study, we present entomological and WNV/USUV detection data from two distinct regional units (RUs, NUTS3 level) of the Attica region (NUTS2 level) in Greece. The epidemiological profile based on previous transmission periods and the different environmental types, wetland and urban, were the reasons for the RUs selection for the surveillance program to be performed. The current study was part of the surveillance program in Attica region during the period 2017–2018.

The current program comprised of two axes with the following objectives:

(i) Monitoring and recording of mosquitoes’ species and population densities in the RUs under study; and
(ii) detection and monitoring of the circulation of WNV and USUV in collected *Cx. pipiens* s.l. for possible co-circulation.

This manuscript aimed to highlight the importance of implementing surveillance programs for the prompt detection of viruses’ circulation in mosquito populations and present, for the first time, surveillance data for USUV in mosquitoes.

## 2. Materials and Methods

### 2.1. Study Area

**Figure 1:**
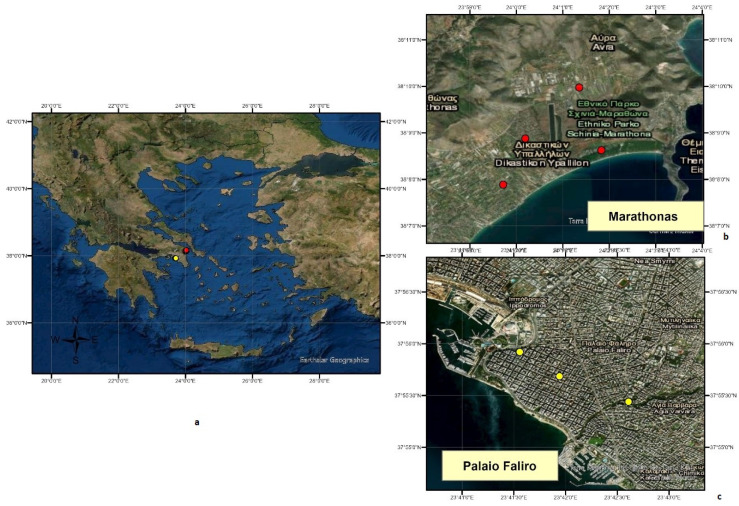
Geographical distribution of BGS traps with a schematic representation of the regional units participating in the research program, 2017–2018. (**a**) map of Greece with locations of surveyed Regional Unit Areas; (**b**) Regional Unit of Marathonas-Schinias (with red dots the sampling locations); (**c**) Regional Unit of Palaio Faliro (with yellow dots the sampling locations).

Two out of eight distinct RUs of Attica comprised the main study area, where traps were installed (Figure 1). The selection of the two RUs was based on the different environmental types, namely urban (UR) and wetland (WT) areas, as described in Table 1. Depending on the research coverage area in each RU, the corresponding number of traps was placed. Additionally, the epidemiological profile of the selected RUs of Attica played an important role concerning the risk for the residents, due to past WNV human infections in both RUs and previously recorded malaria cases in the East Attica Sector being reported to the NPHO since 2010 [38,63]. Data were collected from June 2017 to December 2018.

The region of Attica, “Attiki” in the Greek language (38.0° N 23.7° E; total area: 3808.10 km^2^; population: 3,828,434 inhabitants (2011 record data) [64], is the main metropolitan region of Greece, located on the eastern edge of the mainland, in Central Greece. The greater area of Attica region includes Athens (the capital of Greece) and Piraeus along with 62 other cities and settlements. It is bordered by the sea, to the east, including the south and southwest, while four mountains, Egaleo, Parnitha, Penteli, and Hymettus, delineate the hilly plain [65].

The selected RUs are as follows:

(i) The RU of Marathonas-Schinias (MS): The wetland area (WT) sector of East Attica (EA) (38°0′ N 23°57′ E, total area: 1513 km^2^; population: 502,348 inhabitants [64]) covers the eastern part of the urban agglomeration of Athens, and also the rural area to its east. It is the only Attica zone with significant agricultural activity, can be considered geographically isolated from the rest of the basin, and is inserted between the Penteli and Hymettus mountains. The selected RU of MS (38°9′ N 23°57′ E; total area: 222.75 km^2^; population: 33,423 inhabitants [64]) is located outside the Athens Basin in the northeast part of the Attica region. The MS area lies 42 km away from the center of Athens. Marathonas is an area of intense agricultural activity, while Schinias is an area of marsh and coastal forest. The National Park of Schinias constitutes the most important coastal ecosystem in Attica [66,67].

(ii) The RU of Palaio Faliro (PF): The urban area (UR) covers the south-central part of the agglomeration of Athens in the South Sector of Attica (SS) (37°54′ N 23°44′ E; total area: 69.4 km^2^; population: 529,826 inhabitants [64]). The selected RU of PF (37°56′ N 23°42′ E; total area: 4.574 km^2^; population: 64,021 inhabitants [64]) is a coastal district, situated on the east coast of the Phalerum Bay of the Saronic Gulf, 6 km southwest of the Athens city center. The seaside area of PF is an important touristic attraction with a seaside promenade, several sports venues, and a marina. The Pikrodafni stream flows into the sea on the border of the RUs of Palaio Faliro and Alimos [68].

### 2.2. Mosquito Collection and Identification

The selection of the mosquito monitoring stations was performed following an on-site visit, and it was based mainly upon ecological and social characteristics, such as urban and rural sites, presence of vegetation and shading, occurrence of humans or livestock as potential hosts for adult mosquitoes, proximity to open sources of fresh or still water, nuisance complaints, and convenience of sampling.

The BG-sentinel trap (BGs) (Biogents AG, Regensburg Germany) baited with CO_2_ and BG-Lure [69], which is considered an effective method for mosquito diversity and abundance characterization [2,18,32], was selected as the main monitoring tool.

The composition of mosquito fauna was investigated by the monitoring system of seven BGs traps that was established in seven selected monitoring stations. In particular, depending on the research coverage area in each RU, four traps were placed in MS area, and three in PF area, respectively (Figure 1). A summary of all sampling data concerning the two studied areas is given in Table 1.

All collected mosquito samples were transferred weekly to the Laboratory of Medical Entomology, of Public Health Policy at the University of West Attica for further analysis. Closed and chilled containers containing dry ice were used for the transportation of samples, under the scope of morphological identification of mosquitoes and molecular detection of viruses (WNV and USUV), respectively.

Mosquitoes’ identification, based on morphological characters, was performed after careful examination under a NIKON SMZ645 Stereo Microscope (Nikon Instruments Inc., Surrey, UK), using appropriate dichotomous keys [70,71,72,73].

Throughout the duration of the study, no male *Culex torrentium* adults were identified regarding the morphological identification of the members of *Cx. pipiens* s.l. complex [70,71]. Adult females were characterized morphologically as *Cx. pipiens* s.l., as the two species are indistinguishable morphologically [70].

Adult mosquitoes that were morphologically identified to belong to *Anopheles maculipennis* s.l. complex were further examined by molecular amplification methods, according to previously described protocols [74,75].

### 2.3. Flaviviruses Survey in Culex pipiens Pools

Screening of *Culex pipiens* s.l. pools was designed to evaluate the possible co-circulation of the two viruses (WNV and USUV) in the same mosquito pool. All collected mosquitoes were maintained under cold chain conditions to preserve the virus viability, pooled according to the collection site, date, species, and sex (up to a maximum of 200 individuals per pool), and stored at −80 °C.

### 2.4. WNV and USUV Detection

A total of 366 *Cx. pipiens* s.l. pools were analyzed for WNV (collected under the current surveillance program). Genetic material (RNA) from mosquito pools was extracted by using the Maxwell 16 Automated Nucleic Acid extraction system (Promega, Madison, WI, USA), according to the manufacturer’s instructions for Maxwell16LEV Simple RNA Tissue kit [13]. A TaqMan Real-Time PCR protocol, specific for WNV lineages 1 and 2 detection, was implemented thereafter [76].

These 366 *Cx. pipiens* s.l. pools were also analyzed for the detection of USUV, with a reverse transcription real-time PCR protocol [77] and for selected samples also with a reverse transcription conventional PCR protocol [78].

Screening of the above-mentioned pools was designed to evaluate the possible co-circulation of the two viruses (WNV and USUV) in the same pool.

### 2.5. Infection Rates

The minimum infection rates (MIR) and maximum likelihood estimation (MLE) were calculated using the PooledInfRate program version 4.0 (available at https://www.cdc.gov/westnile/resourcepages/mosqSurvSoft.html, accessed on 20 August 2021) [13,79]. For each region included in the study areas, the respective MIR and MLE values were calculated per 1000 mosquitoes tested. The MLE has the advantage of considering variations in pool size, while the MIR in a study area assumes the presence of a single positive mosquito in a pooled sample.

## 3. Molecular Methods for Identification of Anopheles Mosquitoes

Anopheles species have been incriminated as vectors for transmission of malaria worldwide [80]. Nevertheless, a common limitation in identifying morphologically related species (i.e., *An. maculipennis* s.l. complex) creates an urgent need for implementation of alternative laboratory approaches.

Unidentified by morphological characteristics, specimens of *Anopheles* spp., as well as subspecies belonging to the *An. maculipennis* complex (cryptic species), were subjected to molecular identification by PCR. The nucleotide sequence variation of the ITS2 ribosomal region (ITS2 rDNA) along with the nucleotide sequence of the mitochondrial gene region I (COI) of cytochrome oxidase, respectively, were used as the main targets of the implemented molecular protocol [81,82,83].

A total of 50 Anopheles adult mosquitoes, the majority of which were morphologically identified, were examined at the molecular level. Representative samples were isolated and sent for sequencing analysis [81,82].

### Data Analysis

A Gaussian Generalized Estimating Equation (GEE) model was used to estimate the number of captures in the two areas, Faliro and Marathonas. GEE analysis was conducted using the package “geepack” [84,85] in R v4.0.0 (R Core Team 2013, R Foundation of Statistical Computing, Vienna, Austria).

## 4. Results

### 4.1. Mosquito Fauna Identification

A total of 46,726 (45,663 ♀♀, 1063 ♂♂) adult mosquitoes were collected in all traps from June 2017 to December 2018. The implemented entomological survey revealed the presence of 15 species, classified in six distinct genera.

According to the results in Table 2 from the GEE analysis for the *Culex* and *Aedes* species collected in both study areas, we observed the following:

Culex: there were significantly less *Culex* captures in Faliro than in Marathonas (*p* = 0.028).

Aedes: the number of captures did not differ significantly between Faliro and Marathonas (*p* = 0.441).

A total of 37.810 (80.92%) individuals were captured in the MS area, corresponding to six genera and 15 species. Additionally, 8.916 (19.08%) individuals were captured in PF area, corresponding to three genera and four species, respectively. Three of the captured species, namely *Anopheles sacharovi*, *Culex pipiens* s.l., and *Aedes albopictus,* are of major medical importance. Furthermore, *Cx. pipiens* s.l. (88.25%) and *Ae. albopictus* (8.05%) were by far the most abundant from all the collected species (Table 3).

A total of 531 adult sampling collections (MS, n = 295; PF, n = 236) from established BGs traps were examined from both studied areas, as described in Table 1. Mosquito collection was carried out by 495 sampling collections (MS, n = 277; PF, n = 218), while 36 BGs (MS, n = 18; PF, n = 18) were problematic due to either technical failure that occurred while in operation or without catches, possibly due to the effectiveness of the local mosquito control programs conducted during the transmission period or due to unstable weather conditions, mainly in winter.

The observations regarding the adult sampling collections of the four BGs traps in the MS study area showed differences regarding species abundance, richness, and diversity. Comparing the findings from the four BGs traps in the MS area, we observed the largest numbers of mosquitoes were collected in the MS1 (33.6% of total MS catches) and MS4 (40.5%) traps, followed by MS2 (15.4%) and MS3 (10.5%), while the variety of species was enriched in the MS3 and MS4 traps, respectively (Table 3). Regarding the adult sampling collections of the three BGs traps in PF area, no differences were observed concerning the species diversity. A large number of mosquitoes collected in the PF1 (47.35% of total PF catches) trap were followed by the PF3 (38.72%) and PF2 (13.93%) traps, respectively (Table 3). Taking into consideration that the urban habitats contained more densely human-populated areas than the rural habitats, which had a higher density of livestock, we concluded that species richness and diversity recorded in the surveyed municipalities were within the expected range [32].

**Figure 2:**
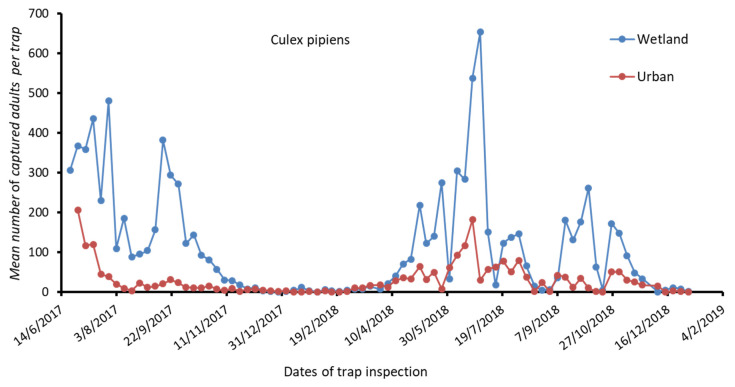
Mean number of *Cx. pipiens* adults captured in the wetland and in the urban study area per trap.

**Figure 3:**
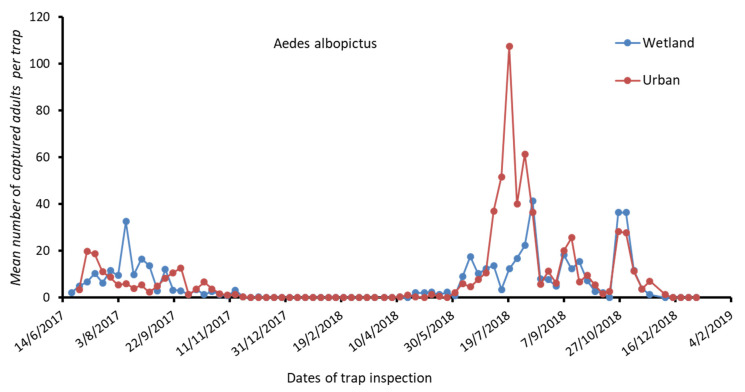
Mean number of *Ae. albopictus* adults captured in the wetland and in the urban study area per trap.

**Figure 4:**
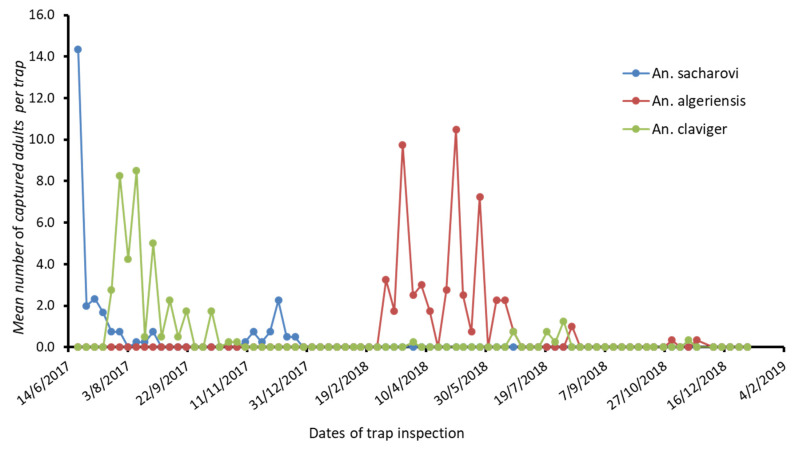
Mean number of the Anopheles adults captured in the wetland study area per trap.

The results for *Cx. pipiens* s.l. and *Ae. albopictus* populations’ fluctuations per week, from June 2017 to December 2018, concerning the surveyed RUs of MS and PF are presented in Figure 2 and Figure 3, respectively. The results for *Anopheles* spp. population fluctuations per week, for the studied period concerning the surveyed RU of MS, are presented in Figure 4.

High numbers of *Cx. pipiens* s.l. were observed in the MS RU from June to September 2017, while in 2018, the population reached a peak in June and then remained relatively low the following months (Figure 2). In the RU of PF, the populations of *Cx. pipiens* s.l. were kept low during both years of entomological surveillance (Figure 2).

A gradual increase in *Ae. albopictus* population was recorded in the MS area since June, reaching a peak in August, followed by a gradual decline in 2017, while populations remained in low numbers in the year 2018 (Figure 3). In the PF area, relatively low numbers of *Ae. albopictus* were recorded in 2017, while there was a peak in July 2018, followed by a gradual decline in the upcoming months (Figure 3).

Of particular importance was the presence of *An. sacharovi* collected in the MS RU, showing an increase in the population in June 2017, then a gradual decrease of catches the following months, and a slight increase was observed in December 2017. In 2018, the catches of the above species were zero during the period of collection (Figure 4). There were no collections of *An. algeriensis* in 2017; this species appeared in the area from March 2018, recording high numbers in April, May, and June, and reaching the peak in May; nevertheless, the population decline from July onwards (Figure 4). *An. claviger* was first collected in July 2017, reaching peak capture rates in August, and then gradually declined in the upcoming months. In 2018, the mosquito populations were kept in low numbers, and a few catches were recorded during the summer months (Figure 4).

### 4.2. Flaviviruses Detection

Of the adult female *Cx. pipiens* s.l. captured, a total of 41.050 (MS, n = 34.358; PF, n = 6.692) were examined in pools for the presence of WNV and USUV. *Cx. pipiens* s.l. adults were treated as a single entity, without determining the relative composition of molestus and pipiens forms.

Out of the 366 mosquito pools tested, a total of 38 (10.4%) tested positive for WNV, including 30 positive samples in MS and 8 positive samples in PF, respectively (Table 4).

The 366 mosquito pools tested for USUV were found to be negative (Table 4). One single pool of 200 *Cx. pipiens* s.l. collected in MS in June 2018 was suspected to be possibly USUV positive (low signal upon real-time PCR assay). A reverse transcription conventional PCR protocol was performed for further analysis. The sample produced a PCR product of low intensity (faint band), which did not contribute much to resolving this issue. The PCR product was subsequently sent for sequencing analysis; however, due to the possible reduced concentration of DNA in the sample, the results were inconclusive and therefore the sample was not confirmed as a positive one. WNV screening in the same sample showed that it was positive for WNV.

#### Infection Rates

**Figure 5:**
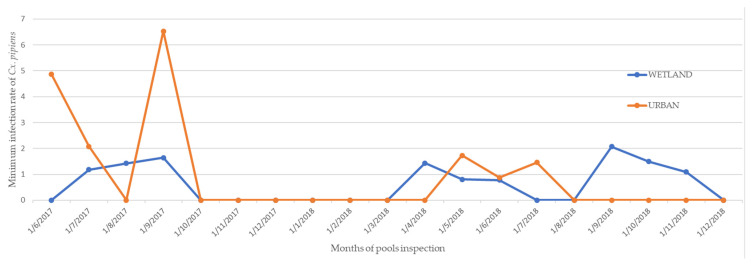
Minimum infection rate (MIR) of *Cx. pipiens* adults captured in the wetland and in the urban study area per month.

In the present study, MLE values both for the wetland and the urban area were almost zero (Table 4), suggesting very low circulation of the virus in the study areas, which is in accordance with the human WNF cases recorded (no cases for 2017 and three cases in the MS area in 2018) [38]. MIR was calculated by extrapolation from the real-time PCR results (the total number of positive pools in the area/total number of mosquitoes sampled in this area × 1000) and is presented in Figure 5. MIR rates indicate that the peak in WNV-infected mosquitoes coincides with high numbers of *Cx. pipiens* populations.

### 4.3. Anopheles Specimens’ Molecular Identification

The species *Anopheles maculipennis* s.l., *Anopheles sacharovi,* and *Anopheles algeriensis* were identified by PCR and RFLP analysis. Further analysis of the sequencing chromatograms in the ITS2 gene identified *An. maculipennis* s.l. and *An. sacharovi* species in eight of the nine tested samples, with the associated traps located in the marsh and the rural environment, respectively. Species identification based on PCR amplification of the COI gene following sequencing of the 522 bp fragment revealed the presence of *An. algeriensis* and *An. sacharovi*, with the relevant traps located in marsh, rural, and semi-arid areas, respectively.

## 5. Discussion and Conclusions

The epidemiology of WNV and USUV has changed dramatically over the past two decades [28,47]. Recent data showed that strains detected in humans, horses, birds, and mosquitoes mainly belong to WNV lineage 2, including the Greek WNV strains detected during 2010–2018 [30,31,39,86,87,88,89]. Various USUV lineages are co-circulating in Europe, the majority of strains are related to European USUV lineages [49,52,53,54,90], although some reports indicate the presence of African USUV lineages as well [43,61,91]. USUV Europe 2 lineage is the most prevalent genetic lineage detected in birds, mosquitoes, and humans, while Europe 3 and 4 and Africa 2 and 3 lineages were detected in mosquitoes [47].

Vector competence plays an important role in vectorial capacity helping in identifying species that might be important contributors to flaviviruses transmission, implementing control measures to reduce the potential of WNV/USUV transmission [28,42], and indicating the possible role of supporting the spread of WNV/USUV during winter [20,53,88,92,93,94,95].

In this study, mosquito screening for WNV showed that the majority of positive samples for WNV were detected in the East Attica, followed by the South Sector of Attica (Table 4). In the RU of PF, WNV-positive pools in mosquitoes were detected in both years, while in the study of Bisia et al. (2020) [18], no positive pool was detected in 2018. The region of MS has a warm temperate climate with hot dry summers and mild winters and displays characteristics to sustain WNV transmission cycles [96]. Due to its ecological and geographical features, this region is considered a risk area for flavivirus transmission [67].

The higher diversity and abundance of mosquito fauna were observed in Marathonas (intense agricultural activity) and Schinias (swamp and coastal forest), confirming similar remarks from previous studies [67,97]. The spatiotemporal monitoring of land cover changes studied by Gaitanis et al. (2015) [66] in the RU of MS showed a reduction of the areas covered by semi-natural and agricultural and cover types (forests, wetlands, shrublands, and cultivated fields) and the increase of urban and mixed areas during the last 60 years. According to the results of the study and records from the resident population, the mosquito nuisance is serious from early spring onwards [97].

Regarding data on the WNV circulation in equids and birds, according to the Ministry of Rural Development and Food, no cases were detected in 2017 in the Attica region. For 2018, confirmed WNV cases in equids were recorded in West Attica and East Attica RUs, and canary birds that were positive for the virus were also detected in the Athens West Sector RU [98].

For 2018, Greece reported 317 WNV infections and 51 deaths, representing 20% of total EU cases and displaying a 6,6 higher rate than in 2017, with 48 human cases being recorded. Regarding the areas of the present study, 11 and 14 human cases were recorded in the South Sector of Athens and the East Attica RUs, respectively, with a total number of 160 cases being recorded in the Attica region for 2018 [99].

The installation and circulation of WNV in Greece is a fact, while extensive studies have been performed on its circulation since 2010 [10,11,12,14,18,23,30,33,34,35]. However, predicting periods and circulation areas of the virus are difficult due to complex interactions of multiple involved factors [23,38,100]. It is noteworthy that, although outbreaks occurred in humans every year apart from the 2015–2016 period [18,37,38,88,100], positive *Cx. pipiens* s.l. pools were detected in different areas of the country [9,13,14,96].

Minimal infection rates of *Cx. pipiens* adults for WNV in both study areas were in accordance with the mosquito population density, and the low infection rates detected are consistent with the low human case rates observed. No human cases were recorded for 2017 and three cases were detected in the wetland area in 2018 [38,99].

The molecular identification of *Anopheles* spp. proved to be a useful tool for supporting morphological identification. This molecular approach using two genetic markers increased taxonomic resolution helped to identify damaged specimens and to distinguish species within a complex. A deeper study on the molecular identification of the Anopheline mosquito complex is required [3], as many of the Anopheles species in the MS area are malaria vectors [96], and indigenous cases of malaria have been recorded in East Attica the years 2009, 2010, 2011, 2012, and 2015 [63]. *An. maculipennis* s.l. is a potential vector of malaria, and it has been considered as an important vector in the past for the spread of this disease in various regions of Greece. *An. sacharovi* is considered to be the principal vector of malaria, from all subspecies of the *An. maculipennis* s.l. complex for Mediterranean countries and of course for Greece [67,75,83]. *An. claviger* is a potential vector of malaria, although its medical significance is not considered to be great for our country. Relatively in high numbers, *An. algeriensis* is a common and very abundant species in the area, captured in all four traps, and although it can easily be infected with malaria plasmodium, it is considered a secondary vector due to its exophily [70].

The findings of this study revealed different assemblages of mosquito species in each targeted RU. Regarding the selected RUs, there were significant differences in many of their ecological characteristics and that was the main factor for their selection. All of the species recorded in this study were collected in the MS RU, while in the PF RU, the main collected species were *Cx. pipiens* s.l., *Ae. albopictus,* and *Cs. longiareolata*, which is in accordance with the study of Bisia et al. (2020) [18]. In both surveyed RUs, *Cx. pipiens* s.l. and *Ae. albopictus* were by far the most abundant species.

This study also provides baseline information and acts as a starting point for further investigation of USUV circulation. With the continuing spread of USUV since 2001 in neighboring countries of Greece [42,47,58], it is important to monitor both viruses before the possible occurrence of an epidemic. According to the epidemiological model, Greece belongs to the areas where the USUV can be transmitted, causing a possible epidemic. It mainly indicates areas in the north of the country, for two possible reasons. First of all, in these areas, especially in the river deltas where the number of mosquitoes has increased, migratory birds appear to have stopped moving from Europe to Africa [101], and secondly, in Northern Greece, the only case so far with antibodies to the virus has been recorded [62].

In the present study, samples from Attica for the years 2017–2018 were found negative for USUV. However, despite the limitations that emerged for confirming one possibly positive sample, USUV and WNV co-circulation cannot be excluded in the future. Up to date, no cases of USUV have been reported in humans or equids in Greece, suggesting that there is no circulation of this virus or, at least, its prevalence is very low. Given the knowledge we have from other relatives of flaviviruses, such as the WNV, the risk of causing even more outbreaks in areas endemic to the USUV or in new ones that have not yet spread are quite high. It must be noted that USUV might be misdiagnosed as WNV when the diagnosis is based only on antibody detection, without testing by PCR or neutralization assays, due to cross-reactivity in serology [42,60,102].

Further investigations of both viruses could provide answers to our suspicions about both the USUV circulation in our country and the interaction with its related flavivirus. Furthermore, entomological surveillance activities should be extended to Attica, especially urban ones, as USUV appears to be equally transmitted in urban and rural areas, in contrast to WNV, where higher transmission rates are recorded in rural areas [26]. Virus surveillance within the native mosquito populations offers an opportunity to detect a virus before the emergence of disease in the susceptible host population [103].

A general comment to be made refers to the fact that Anopheles species identification exclusively by morphological features often presents difficulties, highlighting the necessity for implementation of a targeted molecular protocol to the species level [104]. In our case, Anopheles mosquitoes collected in the Marathonas-Schinias area created a need for the development of a special molecular protocol. In this manuscript, we aimed to highlight that the implemented combined research methodology proved to be a useful tool for supporting morphological identification. Furthermore, the applied molecular methodology was found to be specific and sensitive, regarding the possibility of finding positive mosquito pools for WNV.

In conclusion, the findings of this study emphasize the need for regular monitoring of the mosquito fauna in all regions of Greece, which will contribute to increasing the current knowledge about the diversity, distribution abundance, and ecology of species that are present in the regions. Related studies on mosquito fauna should be performed in all RUs of the Attica region as data on mosquito population and species distribution would be valuable, in particular on those species that are of zoonotic relevance. The implementation of integrated arbovirus surveillance programs represents a relevant and necessary assessment of the risk of pathogen transmission in a given region, allowing for the establishment of the appropriate preventive measures.

## Figures and Tables

**Table 1:** Summary of data regarding sampling sites, catches, weeks, traps, collections of the RUs of the Attica region participated in the research program, 2017–2018.

Attica Region	Surveyed RU Areas (Abbreviation) and Period	BG-Sentinel Traps (BGs) CO_2_ + BG-Lure	Sampling Location	GPS Coordinates (Decimal Degrees)		Microenvironment Description of Sampling Site	No. of Weeks	No. of Collections (Field/Problematic Collections)	Sampling Frequency
				Latitude	Longitude				
East Attica (EA)	Wetland Area (WT) Marathonas-Schinias (MS) 15/06/2017–28/12/2018	MS1	Schinias	38.147729	24.002428	Private house/outdoor garden with a large number of trees and large green spaces, agricultural area (semi-urban area)	80	74 (80/6)	Weekly
		MS2	Schinias	38.131439	23.995250	Private house/outdoor garden with a large number of trees and large green spaces, agricultural area (semi-urban area)	80	69 (80/11)	Weekly
		MS3	Schinias	38.143819	24.030472	Private house/outdoor garden with a large number of trees and large green spaces, agricultural area (bordered by the marsh)	59	59 (59/0)	Weekly
		MS4	Kato Souli	38.166265	24.022584	Private house/outdoor garden with a large number of trees and large green spaces, agricultural area (rural area)	76	75 (76/1)	Weekly
						Total MS	295	277 (295/18)	
South Sector (SS)	Urban area (UR) Palaio Faliro (PF) 21/06/2017–27/12/2018	PF1	Open Protection Centers for Elderly	37.931997	23.692625	Municipality building/outdoor garden with a large number of trees and large green spaces, urban area	79	77 (79/2)	Weekly
		PF2	City Hall	37.928111	23.699008	Municipality building/outdoor garden with a large number of trees and large green spaces, urban area	79	77 (79/2)	Weekly
		PF3	Rema Pikrodafnis	37.923989	23.710129	Private house/outdoor garden with a large number of trees and large green spaces, urban area	78	64 (78/14)	Weekly
						Total PF	236	218 (236/18)	

**Table 2:** Results from the GEE analysis for the Culex and Aedes species.

	B (95% CI)	Wald χ2	*p*
***Culex***			
Intercept	117.608 (38.523–196.693)	8.50	0.004
Area: Faliro (ref: Marathonas)	−90.582 (−171.388, −776)	4.83	0.028
***Aedes***			
Intercept	6.560 (1.965, 11.154)	7830	0.005
Area: Faliro (ref: Marathonas)	3.038 (−4.685, 10.762)	0.594	0.441

**Table 3:** Species composition and relative abundance (%) in adult mosquitoes trapped in the WT and UR areas of the Attica region that participated in the research program, 2017–2018.

Mosquito Species	Total Number/Species (%)	F ♀	M ♂	Marathonas-Schinias (MS) Wetland Area (WT)					Palaio Faliro (PF) Urban Area (UR)			
				MS1	MS2	MS3	MS4	Total Number/Species (%)	PF1	PF2	PF3	Total Number/Species (%)
*Aedes (Stegomyia) albopictus (Skuse) ^#^*	3762 (8.05)	3041	721	828	685	87	155	1755 (3.75)	991	160	856	2007 (4.3)
*Aedes (Ochlerotatus) caspius (Pallas)*	81 (0.173)	79	2	21	21	6	27	75 (0.16)	0	0	6	6 (0.013)
*Aedes (Ochlerotatus) detritus (Haliday)*	588 (1.25)	580	8	187	105	247	49	588 (1.25)	0	0	0	0
*Anopheles (Anopheles) algeriensis (Theobald)*	209 (0.45)	209	0	22	2	67	118	209 (0.45)	0	0	0	0
*Anopheles (Anopheles) claviger (Meigen)*	160 (0.34)	160	0	39	2	34	85	160 (0.34)	0	0	0	0
*Anopheles (Anopheles) maculipennis s.l. (Meigen)*	2 (0.0042)	2	0	1	0	1	0	2 (0.0042)	0	0	0	0
*Anopheles (Anopheles) sacharovi (Favre)*	93 (0.2)	93	0	43	0	33	17	93 (0.2)	0	0	0	0
*Coquillettidia (Coquillettidia) richiardii (Ficalbi)*	164 (0.35)	164	0	55	0	41	68	164 (0.35)	0	0	0	0
*Culex (Culex) pipiens (Linnaeus)*	41,236 (88.25)	41,050	186	11,480	4989	3416	14,643	34,528 (73.90)	3150	985	2573	6708 (14.35)
*Culex (Culex) theileri (Theobald)*	16 (0.034)	16	0	0	0	0	16	16 (0.034)	0	0	0	0
*Culiseta (Culiseta) annulata (Schrank)*	11 (0.023)	11	0	6	0	3	2	11 (0.023)	0	0	0	0
*Culiseta (Culicella) fumipennis (Stephens)*	5 (0.010)	0	5	0	0	5	0	5 (0.010)	0	0	0	0
*Culiseta (Allotheobaldia) longiareolata (Macquart)*	395 (0.84)	254	141	34	21	18	127	200 (0.43)	81	97	17	195 (0.41)
*Culiseta (Culiseta) subochrea (Edwards)*	3 (0.0064)	3	0	2	0	0	1	3 (0.0064)	0	0	0	0
*Uranotaenia (Pseudoficalbia) unguiculata (Edwards)*	1 (0.002)	1	0	0	0	0	1	1 (0.002)	0	0	0	0
Total	46,726	55,307	1347	12,718	5825	3958	15,309	37,810	4222	1242	3452	8916

^#^ Aedini denomination according to Wilkerson et al. (2015).

**Table 4:** *Culex pipiens* pools tested for West Nile virus and Usutu virus (Nt); the number of West Nile virus- and Usutu virus-positive *Culex pipiens* pools (Np) per surveyed RUs; and the year and maximum likelihood estimate (MLE) of the infection rate values for the urban and wetland areas, Attica Research Program, 2017–2018.

Surveyed RUs	West Nile Virus				Usutu Virus		
	2017	2018	Total No. of Positive/Tested Pools	MLE	2017	2018	Total No. of Positive/Tested Pools
	Np/Nt *	Np/Nt			Np/Nt	Np/Nt	
East Attica/Marathonas-Schinias (wetland area)	18/120	12/147	30/267	0.00010 (95% CL 0.0007–0.0014)	0/120	0/147	0/267
South Sector/Palaio Faliro (urban area)	5/33	3/66	8/99	0.01 (95% CL 0.0006–0.0026)	0/33	0/66	0/99
Total no. of positive/tested pools per transmission year	23/153	15/213	38/366		0/153	0/213	0/366

* Np/Nt = number of positive pools/number of tested pools.
